# Autistic Siblings with Novel Mutations in Two Different Genes: Insight for Genetic Workups of Autistic Siblings and Connection to Mitochondrial Dysfunction

**DOI:** 10.3389/fped.2017.00219

**Published:** 2017-10-12

**Authors:** Barrett J. Burger, Shannon Rose, Sirish C. Bennuri, Pritmohinder S. Gill, Marie L. Tippett, Leanna Delhey, Stepan Melnyk, Richard E. Frye

**Affiliations:** ^1^University of Arkansas for Medical Sciences, Little Rock, AR, United States; ^2^Autism Research Program, Arkansas Children’s Research Institute, Little Rock, AR, United States

**Keywords:** autism spectrum disorder, DEP domain-containing protein 5, neurodegeneration with brain iron accumulation, Rett syndrome, WDR45, whole exome sequencing

## Abstract

The prevalence of autism spectrum disorder (ASD) is high, yet the etiology of this disorder is still uncertain. Advancements in genetic analysis have provided the ability to identify potential genetic changes that may contribute to ASD. Interestingly, several genetic syndromes have been linked to metabolic dysfunction, suggesting an avenue for treatment. In this case study, we report siblings with ASD who had similar initial phenotypic presentations. Whole exome sequencing (WES) revealed a novel c.795delT mutation in the WDR45 gene affecting the girl, which was consistent with her eventual progression to a Rett-like syndrome phenotype including seizures along with a stereotypical cyclic breathing pattern. Interestingly, WES identified that the brother harbored a novel heterozygous Y1546H variant in the DEP domain-containing protein 5 (DEPDC5) gene, consistent with his presentation. Both siblings underwent a metabolic workup that demonstrated different patterns of mitochondrial dysfunction. The girl demonstrated statistically significant elevations in mitochondrial activity of complex I + III in both muscle and fibroblasts and increased respiration in peripheral blood mononuclear cells (PBMCs) on Seahorse Extracellular Flux analysis. The boy demonstrates a statistically significant decrease in complex IV activity in buccal epithelium and decreased respiration in PBMCs. These cases highlight the differences in genetic abnormalities even in siblings with ASD phenotypes as well as highlights the individual role of novel mutations in the WDR45 and DEPDC5 genes. These cases demonstrate the importance of advanced genetic testing combined with metabolic evaluations in the workup of children with ASD.

## Introduction

Autism spectrum disorder (ASD) is a behaviorally defined disorder characterized by social communication deficits along with restricted interests and repetitive behaviors, which affects nearly 2% of children (1). Over the past two decades, the incidence of ASD has increased dramatically, yet, the etiology of ASD still remains largely poorly understood. It is believed that environmental and genetic factors contribute about equally (2) and several predisposing factors such as paternal age, prematurity, prenatal folate, and endocrine abnormalities influence the risk of developing ASD (3).

The persistent concentration on genetic causes of ASD (4) is fueled by the high recurrent risk of ASD in siblings and the association of ASD with well-defined genetic syndromes, such as Down and Turner (5). About 10% ASD cases demonstrate *de novo* or inherited copy number variations, such as microdeletion or microduplication syndromes (5–8). Fragile X accounts for up to 5% of ASD cases, while cytogenetic abnormalities account for another 5% (8). The remaining single-gene defects are attributed to rare genetic disorders such as Rett syndrome, tuberous sclerosis, and PTEN syndrome (5).

The diagnostic yield of whole exome sequencing (WES) varies from 3 to 29% depending on the characteristics of the sample (9). Interesting, the majority of rare mutations in children with ASD, like copy number variations, are *de novo* rather than inherited. In fact, cohort studies have estimated that *de novo* events may be causative in as many as 20% of ASD cases (10). Although genetic abnormalities are believed to drive the increased risk of occurrence of ASD in subsequent siblings, genetic studies suggest that the great majority (~70%) of siblings demonstrate different genetic mutations (11). This again, highlights the case for mutations being *de novo* rather than inherited, with different *de novo* mutations affect members of the same family.

The natural question is how such various different mutation may all cause ASD. Interestingly, many genetic disorders, particularly those associated with ASD, are linked to metabolic abnormalities. For example, mitochondrial dysfunction is documented in many genetic syndromes associated with ASD, including Rett syndrome (12–14), PTEN mutations (15), Phelan-McDermid syndrome (16), 15q11-q13 duplication syndrome (17, 18), Angelman syndrome (19), septo-optic dysplasia (20), and Down syndrome (21, 22). Down and Rett syndrome have also been linked to redox abnormalities (23, 24) and Rett Syndrome has been linked to cerebral folate deficiency (25). Thus, it is important to determine if associated metabolic conditions may occur in genetic syndromes as it may represent a common factor that may be driving ASD pathophysiology as well as provides a path for treatments that could potentially improve cognitive and behavioral function.

The genetics of mitochondrial dysfunction in ASD is interesting and potentially unique. Our meta-analysis found that classic mitochondrial disease affects about 5% of children with ASD (26). However, this meta-analysis also found that only about 25% individuals diagnosed with ASD and concomitant mitochondrial disease were reported to have a genetic defect to explain their mitochondrial disease. What is more interesting is that a significant percentage of children with ASD, 30% (26) or more (27, 28), manifest biomarkers of mitochondrial dysfunction, suggesting that many children with ASD may have a novel type of mitochondrial dysfunction, which, perhaps, is related to physiological dysfunction in non-mitochondrial pathways. Alternatively, deleterious environmental agents or heterozygous mutations in nuclear mitochondrial genes could combine with mitochondrial DNA polymorphisms to result in mitochondrial dysfunction in a complex manner that would be difficult to determine clinically (29). Mitochondrial dysfunction is believed to be important in ASD as biomarkers of mitochondrial dysfunction in children with ASD are correlated with ASD severity (30, 31) and, recently, we demonstrated that abnormal mitochondrial function was related to worse repetitive and stereotypical behavior in a cell line sibling study of ASD (32). In addition, patients with mitochondrial dysfunction have the potential to response to treatment even if the mitochondrial dysfunction is secondary to a non-mitochondrial defect (33).

In this case report, we present a 7-year-old female with a history of regressive-type ASD and her 6-year-old brother with a similar history. The girl developed a Rett syndrome phenotype but genetic testing for Rett and Rett-like syndrome genes was negative. Subsequent WES revealed a *de novo* c.795delT mutation in the WDR45 gene, a previously unidentified pathological mutation. WES revealed a novel mutation in the DEP domain-containing protein 5 (DEPDC5) gene for the boy. This report highlights the Rett-like presentation of the WDR45 gene and the fact that siblings can have different genetic causes of ASD. In addition, this is the first report to demonstrate the parallel between mitochondrial alterations in a WDR45 patient and those associated with Rett syndrome and mitochondrial abnormalities associated with DEPDC5. To this end, we measured mitochondrial function using several techniques, specifically enzymology of fibroblasts, muscle, and buccal tissue, and high-throughput respirometry of immune cells.

## Materials and Methods

### Regulatory Approval

The study was conducted under two protocols approved by the Institutional Review Board at the University of Arkansas for Medical Sciences (Little Rock, AR, USA). One protocol specifically provided permission to investigate data collected as part of clinical evaluations while the second protocol specifically addressed obtaining samples for biochemical analysis as well as developmental and behavioral testing. Parents of participants provided written informed consent.

### Skin and Muscle Enzymology

Muscle and/or skin biopsy can provide important information regarding mitochondrial function (34). A skin biopsy provides fibroblasts for both electron transport chain (ETC) and fatty-acid oxidation pathway testing (28). ETC activity in multiple tissues can examine homogeneity and heterogeneity in mitochondrial activity across tissues (4). Thus, if a muscle biopsy is performed, a skin biopsy to obtain fibroblast is usually performed simultaneously.

Functional ETC studies were conducted on the muscle and fibroblasts derived from the skin (Baylor Medical Genetics Laboratory, Houston, TX, USA) (35). The assay is conducted in duplicate and the average is reported. Fibroblasts derived from skin were also incubated with d3-palmitate and l-carnitine in duplicate for 72 h to determine function of the beta-oxidation pathway (Baylor Institute of Metabolic Disease, Dallas, TX, USA) (36).

Assays for determining ETC complex activity using spectrophotometry are derived from the methods of Kirby et al. (35). In brief, the absorbance, a specific wavelength of light, can be used to determine enzyme activity. For example, the reduced form of NAD (NADH) strongly absorbs UV light at 340 nm whereas the oxidized form of NAD does not absorb light at this wavelength. Thus, for complex I, activity can be inferred from the rate of change of UV light at 340 nm. To determine how much of the rate of change in absorbance is the result of complex I activity, a specific enzyme inhibitor, such as rotenone, is added to determine the change in the rate of absorbance. Complex III cannot be measured alone using this method, so it is measured in combination with ETC Complex I or II. For clinical evaluation, these measurements are usually presented as a percent of normal in order to allow activity to be compared across complexes on the same scale since the absolute absorbance differs widely from complex assay to complex assay and from tissue to tissue. However, only providing a percent of normal measure makes it difficult to compare activity values to the normal range. Thus, we present activity for each ETC complex measurement both as a percent of normal and as compared to the normal control range for that specific tissue and ETC complex. To statistically compare the patient samples to the control population, we calculated the *z*-score using the control population SD and mean and calculated the associated probability of the patient’s value being in the normal distribution. Only significant values are reported.

### Buccal Enzymology

We measured activity of complex I and IV as well as citrate synthase (CS) using a validated buccal swab procedure (37), which has been used previously to measure mitochondrial function in children with ASD (38–40) and other developmental (41) and neurologic (42) disorders. Buccal cells were collected using Catch-All Buccal Collection Swabs (Epicentre Biotechnologies, Madison, WI, USA). Four swabs were collected by firmly pressing a swab against the inner cheek while twirling for 30 s. Swabs were clipped and placed in 1.5 mL microcentrifuge-labeled tubes and placed on dry ice for overnight transportation to the laboratory.

Buccal extracts were prepared using an ice-cold buffered solution (Buffer A, ABCAM) containing protease inhibitor cocktail and membrane solubilizing non-ionic detergent and cleared of insoluble cellular material by high-speed centrifugation at 4°C. Duplicate aliquots of the protein extract were analyzed for protein concentration using the bicinchoninic acid method (Pierce Biotechnology, Rockford, IL, USA). Samples were typically stored at −80°C for up to 1 week prior to enzymatic analysis.

Dipstick immunocapture assays measured ETC Complex I activity using 50 µg extracted protein (37, 42). Signals were quantified using a Hamamatsu immunochromato reader (MS 1000 Dipstick reader). Raw mABS (milliAbsorbance) results were corrected for protein concentration and data were expressed as percentages of the values obtained with control extracts run on the same assay. ETC Complex IV and CS activity were assessed using standard spectrophotometric procedures in 0.5 mL reaction volume.

Specific activities of respiratory complexes were initially expressed as nanomoles per milligram protein per minute and normalized to CS activity levels. The use of activity ratios is well established and provides a much narrower range of control values as compared to activities expressed on the basis of protein content.

Controls for the buccal swab assay included 106 healthy individuals without neurological disease who have been described and used in previous studies (37). Controls ranged in age from 2 to 49 years of age [mean (SD) 10.2 years (8.4 years)] with 52 (49%) being females. In a previous report, it was found that there was no correlation between protein activities and age and no difference in protein activities across ethnicity or race in both controls and previously studied patients (37).

To statistically compare the patient samples to the control population, we calculated the *z*-score using the control population SD and mean and calculated the associated probability of the patient’s value being in the normal distribution. Only significant values are reported.

### Respirometry-Based Assays

We also present bioenergetic data from peripheral blood mononuclear cells (PBMCs) obtained using a state-of-the-art Seahorse 96 XF Analyzer (Seahorse Bioscience, Inc., North Billerica, MA, USA) that measures oxygen consumption rate in real time in a 96-well plate. This analyzer evaluates a wide range of intact living cell types (43, 44) and can detect ETC (45), glycolytic (46), and fatty-acid oxidation (47) abnormalities. The assay measures several key parameters: (a) adenosine triphosphate (ATP)-linked respiration, (b) proton leak respiration, (c) maximal respiratory capacity, a parameter that is sensitive to deficits in mitochondrial biogenesis, mtDNA damage and/or inhibition of ETC function, and (d) reserve capacity, a parameter that determines the threshold at which bioenergetic dysfunction occurs (48). The assay measures four samples in parallel to obtain quadruplicate measurements for each index of bioenergetics for each patient.

Ethylenediaminetetraacetic acid (EDTA) vacutainers of whole blood were centrifuged to separate plasma. Plasma was removed and replaced with room temperature wash buffer containing Ca^+2^/Mg^+2^-free PBS with 0.1% BSA and 2 mM EDTA. Diluted blood was then layered on top of Histopaque-1077 (Sigma Aldrich, St. Louis, MO, USA) and centrifuged at 400 × *g* for 30 min at room temperature. PBMCs were washed twice with wash buffer and counted using a hemocytometer. PBMCs were placed in assay media (unbuffered RPMI supplemented with 1 mM pyruvate, 2 mM glutamate, and 25 mM glucose) and plated at approximately 4 × 10^5^ cells per well of a poly-d-lysine-coated XF96 well plate for the assay described above. Assays were run in quadruplicate. Runs with clear measurement probe failure or reagent injection failure were eliminated.

To obtain normative values, PBMCs from 17 typically developing siblings of children with ASD were evaluated with same mitochondrial assay. This control population was 47% females and ranged in age from 2 years 9 months to 15 years 10 months of age.

To statistically compare the patient samples to the control population, 71 measurements derived from these controls (2 to 4 replicates per control with 2 control subject providing two different samples at least 6 months apart) were compared to the individual patient sample values using a heteroscedastic *t*-test with a two-tailed alpha probability ≤0.05.

### Whole Exome Sequencing

Whole exome sequencing was performed by Gene Dx (Gaithersburg, MD, USA) using standard techniques (49). Briefly, Agilent SureSelect XT2 All Exon V4 kit (Agilent Technologies) was used to target the exon regions of the genome from genomic DNA derived from whole blood. Exon regions were sequenced using the Illumina HiSeq 2000 with massive parallel sequencing and 100 bp paired-end reads. DNA sequence was mapped to the human genome build UCSC hg19 reference sequence. Targeted coding exons and splice junctions of the known protein-coding RefSeq genes demonstrated a mean depth of coverage of 106× with 97.8% of the targeted regions covered at least 10×. XomeAnalyzer (GeneDX, Gaithersburg, MD, USA) was used to evaluate sequence changes in this individual compared to other sequenced family members. All identified sequence variants in the proband and relative samples were confirmed by conventional di-deoxy DNA sequence analysis.

### Mitochondrial DNA Sequencing

Mitochondrial DNA sequencing and deletion testing was performed by Gene Dx (Gaithersburg, MD, USA). In brief, the entire mitochondrial genome was amplified and sequenced using a solid-state sequencing by-synthesis process. DNA sequence was assembled and analyzed in comparison with the revised Cambridge Reference Sequence and mutations and polymorphisms listed in the MITOMAP database (http://www.mitomap.org) were reported.

### Developmental Assessments

The Clinical Evaluation of Language Fundamentals, a test that is routinely used to assess language impairment in children with ASD, was used to assess language ability. Standardized scores are reported compared to children that are the same chronological age with average performance considered 100 and a SD of 15 points. The Vineland Adaptive Behavior Scale (VABS), a parent-based interview, was used to measure developmental abilities. This VABS is divided into four primary domains consisting of: communication, daily living skills, socialization, and motor skills. These tests are routinely used in a clinical setting to assist in the diagnosis of ASD. Standardized scores are reported compared to children that are the same chronological age with average performance considered 100 and a SD of 15 points.

## Results

We report on a female (case 1) and a male (case 2) set of siblings with ASD who were referred to our clinic for a comprehensive medical assessment. The phenotype presentation and laboratory data are described separately.

### Phenotype Presentation

Case 1 was born *via* an unremarkable, full-term vaginal delivery. Atypical development was noticed at 6 months of age when she was using her left side more than her right but she was sitting without support at that time. However, at 1 year of age, she was not able to get herself to a sitting position, leading to referral to the early intervention program. At 18 months, she was able to cruise, 20 months, she was able to stand independently, and at 23 months, she could walk independently. She demonstrated language and communication delays with her first word not emerging until 15 months and lacking spontaneous pointing. At 15 months, she was diagnosed with global developmental delay.

At the age of 15 months, she experienced a febrile seizure. An EEG at that time showed bilateral occipital slowing. A subsequent brain magnetic resonance imaging (MRI) revealed parenchymal loss and ventriculomegaly. She was chronically constipated with an easily upset stomach. Previous lumbar puncture demonstrated a 5-methyltetrahydrofolate (5-MTHF) level of 59, which is low normal.

Family history was significant for a younger brother with developmental delay and regressive-type ASD (case 2), a father with periodic limb movements during sleep and migraine headaches and a mother with sleep apnea and narcolepsy. Several members on the father’s side of the family were diagnosed with attention-deficit disorder without hyperactivity.

On physical examination, she was a tired-appearing young, nonverbal girl with weight at the 45th percentile, height at the 18th percentile, and a head circumference at the 50th percentile. Her cranial nerves were grossly intact with bilateral ptosis. Gross motor exam identified global hypotonia. Deep tendon reflexes were diminished in the lower extremities. No ataxia or tremors were noted. Of note, the patient appeared to skip when attempting to run.

Given the presentation of a developmental-delay with multiple organ involvement [gastrointestinal (GI), neurological, and muscular skeletal], we suspected a mitochondrial problem and proceeded with a workup for mitochondrial disease (see Laboratory Data). Calculated Morava et al. criteria for this patient was four points consistent with possible mitochondrial disorder and included developmental delay (1 point) and seizures (1 point) as well as GI abnormalities (1 point) and recurrent/familiar inheritance (1 point). Given the possible involvement of the mitochondria, a trial of a mitochondrial cocktail was initiated including Co-Enzyme Q10 and carnitine and folinic acid for possible cerebral folate deficiency as many children with ASD respond to folinic acid supplementation with low normal 5-MTHF concentrations.

The patient did well on the mitochondrial cocktail with improvements in interactions, language, and development. However, a neurodevelopmental regression occurred characterized by multiple cyclic episodes of fatigued associated with aberrant behavior and then returning to baseline. These episodes also included teeth grinding and cyclic changes in breathing characterized by periods of hyperventilation followed by hypoventilation, not unlike the breathing pattern of children with Rett syndrome. The mitochondrial cocktail and all other non-essential treatments were discontinued to determine if these new episodes could have been iatrogenic but the episodes continued, leading to a more extensive workup including a muscle biopsy (see Laboratory Data).

At the time of the evaluation, language and developmental testing demonstrated severe impairment. Expression, receptive, and core language scores were 47, 45, and 40, respectively (scaled scores) while developmental assessment on the VABS was 54, 57, 59, and 56 on the communication, daily living skills, socialization and motor skills subscales, and 58 on the adaptive behavior composite.

Interestingly, she was prescribed clobazam for her active EEG. 10 mg of clobazam twice a day resolved the stereotyped breathing episodes. When an attempt was made to wean the clobazam, the breathing episodes returned and were then again controlled once the clobazam was increased again.

Case 2 was a boy born, the normal product of a full-term pregnancy. His milestones were normal during early infancy but, at 1 year of age, decreased reciprocity was noticed and he had no discernible speech. This improved with early intervention program therapy, but this progress regressed by 2 years of age. At the age of three, he was noticed to have problems in school due to hyperactivity. He was diagnosed with ASD later the same year, albeit with a seemingly milder symptoms than his affected sister.

The past medical history was significant for constipation, severe gastroesophageal reflux disease, and sleep-disordered breathing. There was no history of headaches or visual disturbances.

On physical exam, his height was at the 80th percentile, his weight was at the 66th percentile, and his head circumference was macrocephalic at the 98th percentile. He was slightly hypotonic with mildly decreased reflexes in all extremities. Cranial nerves were intact globally.

Calculated Morava et al. criteria for this patient was six points consistent with probable mitochondrial disorder and included developmental delay (1 point), regression (1 point) as well as GI abnormalities (1 point), and recurrent/familiar inheritance (1 point) along with elevation in lactate (2 points). Given the signs of probably mitochondrial disorder, a trial of a mitochondrial cocktail was initiated including coenzyme Q10 and carnitine. The patient did have some improvement in function with more interaction and participation in therapy.

At the time of the evaluation, language and developmental testing demonstrated severe impairment. Expression, receptive and core language scores were 50, 49, and 45, respectively (scaled scores) while developmental assessment on the VABS demonstrated 57, 66, 53, and 75 on the communication, daily living skills, socialization and motor skills subscales, and 59 on the adaptive behavior composite.

### Laboratory Data

For case 1, the workup for mitochondrial dysfunction demonstrate a normal fasting lactate (0.8 mmol/L; nl ≤ 1.3) and alanine-to-lysine ratio (1.6, nl < 2.5) but an elevated pyruvate on two occasions (0.12 and 0.09 mmol/L; nl ≤ 0.08). Elevations were also noted in several short-chain (C2 27.78, nl ≤ 16.56; C5:1 0.07; nl ≤ 0.04), medium-chain (C6 0.14 mcmol/L, nl ≤ 0.12; C8:1 0.65, nl ≤ 0.61), and long-chain (C14 0.07, nl ≤ 0.05; C18 0.05, nl ≤ 0.04) acylcarnitines with normal free and total carnitine levels.

**Figure 1:**
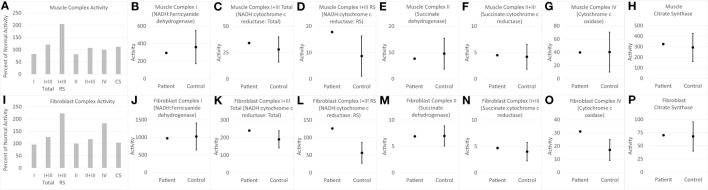
Measurements of electron transport chain function for child with WDR45 mutation in muscle **(A–H)** and fibroblasts **(I–P)**. The left most panel for muscle **(A)** and fibroblast **(I)** samples provide the measurements of each complex as percent of normal, which is the standard clinical presentation of values. Measurement of activity of each complex is then displayed in relation to the normal range of values. The mean and 95% limits of the normal distribution are displayed so that the activity measurements that are clearly outside of the normal range can be seen. Activity values are not normalized to citrate synthase (CS) since CS is close to control average values. RS, Rotenone Sensitive.

**Figure 2:**
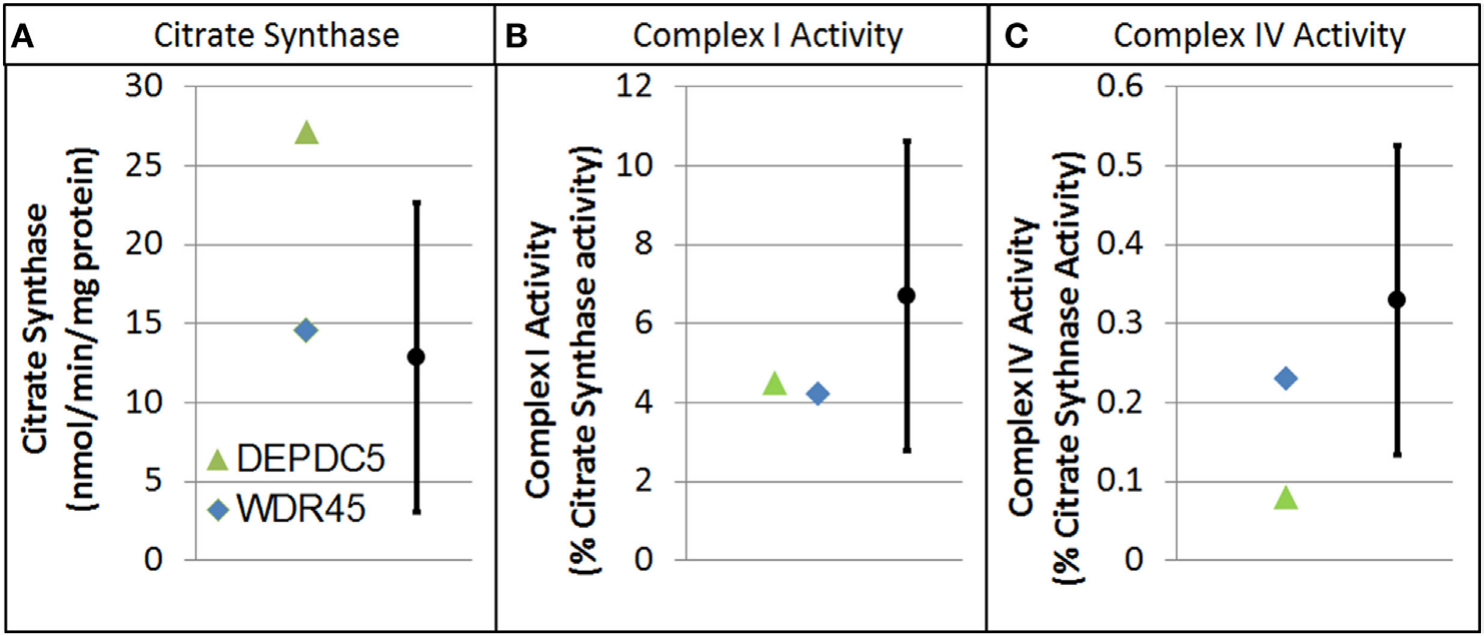
Buccal cell mitochondrial electron transport chain complex and citrate synthase (CS) activity for patients with genetic changes in the WDR45 and DEP domain-containing protein 5 (DEPDC5) genes. As described in the text, values are normalize to CS. **(A)** CS. **(B)** Complex I activity. **(C)** Complex IV activity.

Electron transport chain studies on muscle and skin biopsies demonstrated significant elevations in several complexes (Figure 1). In muscle Complex I + III, Rotenone Sensitive activity was significantly elevated (204% of normal; *z* = 2.33, *p* < 0.01). Fibroblasts also demonstrating elevations in the activity of several complexes, specifically Complex I + III total (126% of normal; *z* = 2.00, *p* = 0.02) and Rotenone Sensitive (222% of normal; *z* = 4.63, *p* < 0.0001) as well as Complex IV (182% of normal; *z* = 3.41, *p* < 0.0005). Fatty oxidation studies revealed no abnormalities. As follow-up to the abnormal mitochondrial laboratory values, we conducted a buccal swab for enzymatic function (Figure 2), which demonstrated a slight reduction in Complex I (61% of normal) and IV activity (70% of normal).

**Figure 3:**
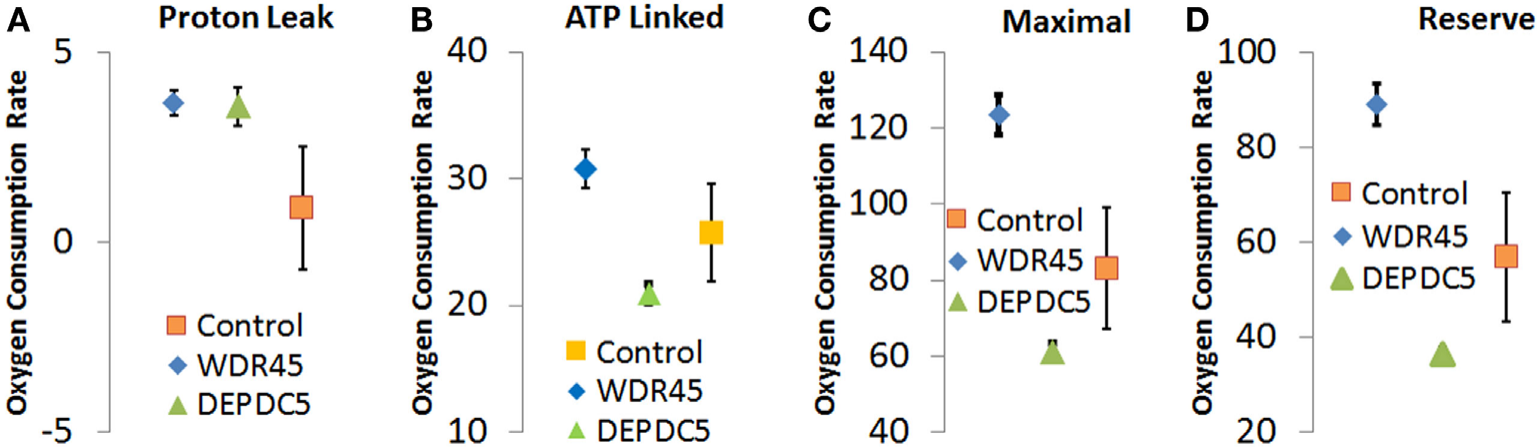
Seahorse Assay for patient with genetic changes in WDR45 and DEP domain-containing protein 5 (DEPDC5) genes. Measurements including **(A)** proton leak respiration, **(B)** ATP-linked respiration, **(C)** maximal respiratory capacity, and **(D)** reserve capacity. Measurements include the patients as well as the control population. Assays were run in quadruplicate and the standard error for the two patients as well as the control population are shown.

Through an IRB approved protocol, we measured mitochondrial function using respirometry and measured development and behavior. As see in Figure 3, Seahorse respirometry measurement of PBMCs demonstrated an statistically significant increase in Proton Leak [511% of normal, *t*(74) = 2.26, *p* = 0.01], ATP-linked respiration [121% of normal *t*(74) = 4.63, *p* < 0.0001], maximal respiratory capacity [154% of normal, *t*(74) = 3.94, *p* < 0.0001], and reserve capacity [166% of normal, *t*(74) = 4.08, *p* < 0.0001].

**Figure 4:**
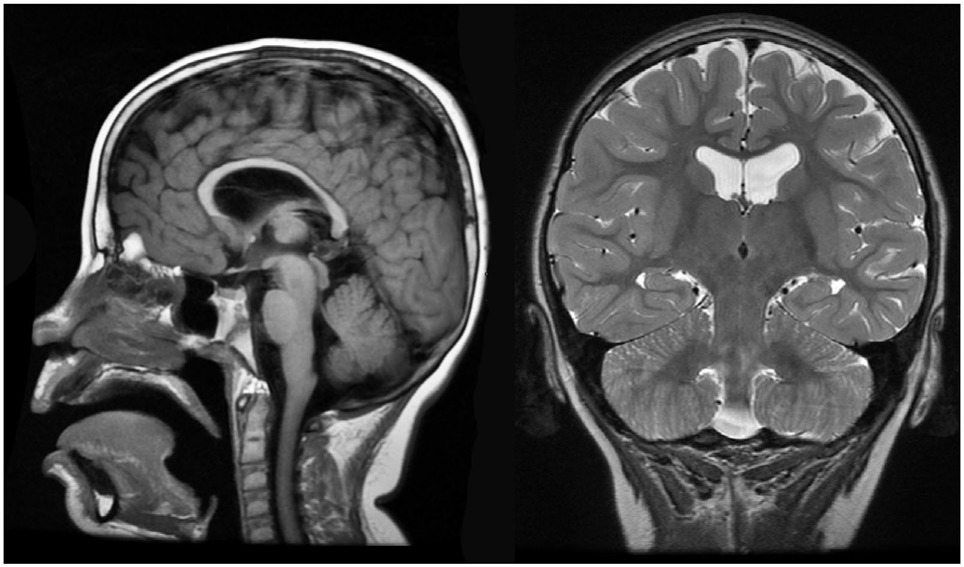
Case 1 (WDR45) high resolution 3T magnetic resonance imaging brain scan. Sagittal T1 fluid attenuated inverse recovery imaging (left) demonstrates thin corpus callosum. T2 coronal imaging (right) demonstrates slight cortical atrophy and slight hippocampal asymmetry.

Brain MRI identified slight cortical atrophy involving the superior frontoparietal convexity bilaterally (Figure 4, right), thinning of the corpus callosum (Figure 4, left and right), and slight hippocampal asymmetry (Figure 4, right). There was also slight paucity of supratentotial white matter bilaterally, particularly along the centrum semiovale, which raised the consideration of a mild degree of periventricular leukomalacia.

**Figure 5:**
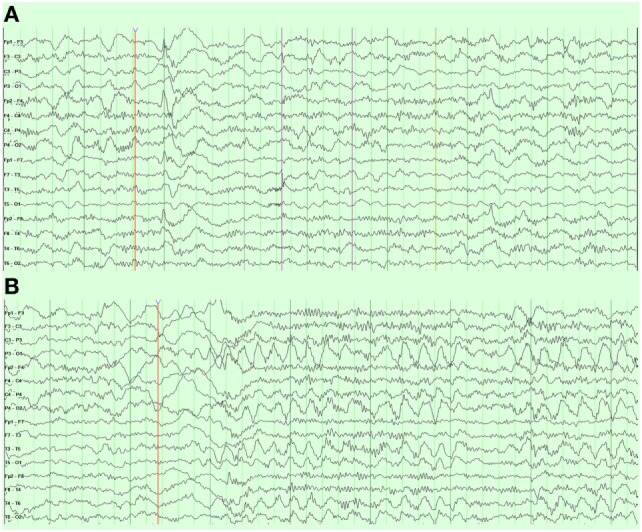
Case 1 (WDR45) electroencephalogram demonstrating **(A)** right frontal spike and wave discharges and **(B)** periodic posterior rhythmic slowing.

An overnight EEG showed diffuse excessive beta activity in wake and sleep, diffuse slowing, intermittent bursts of high amplitude, posterior semirhythmic delta waves intermixed with sharp and spike waves that occurred more often in the left hemisphere (Figure 5A). Additional posterior interictal and epileptiform discharges with occasionally spiking waves were noted (Figure 5B).

Whole exome sequencing revealed that our patient was heterozygous for the *de novo* c.795delT frameshift mutation in exon 10 of the WD repeat domain 45 (WDR45; Entrez ID 11152; UniProt ID Q9Y484) gene resulting in a premature stop codon and loss of normal protein function. The frameshift starting at codon phenylalanine 265 changed the amino acid to a leucine residue, which created a premature stop codon at position 23 of the new reading frame. This variant has not been published as a mutation nor has it been reported as a benign variant in the NHLBI Exome Sequencing Project database of approximately 6,500 individuals of European and African American ancestry. This mutation was not identified in her affected brother, unaffected sister, or either parent. Mitochondrial DNA sequencing and deletion analysis was unremarkable as was a chromosomal microarray (ACL Laboratories, Rosemont, IL, USA).

For Case 2, an overnight EEG revealed bilateral independent epileptogenic foci in the frontal regions occurring as spikes, polyspikes, and spikes followed by high-amplitude slow wave during light sleep. Workup for mitochondrial dysfunction demonstrated an elevation in fasting lactate (2.1 mmol/L; nl ≤ 1.3) and pyruvate (0.14 mmol/L; nl ≤ 0.08) as well as an elevated alanine-to-lysine ratio (2.5, nl < 2.5). Elevations in several medium-chain (C6 0.19 mcmol/L, nl ≤ 0.12; C8:1 0.73, nl ≤ 0.63; C10 0.36, nl ≤ 0.35) and long-chain (C18:2 0.02, nl ≤ 0.01) acyl-carnitines with normal free and total carnitine levels.

As follow-up to the abnormal mitochondrial laboratory values, we conducted a buccal swab for enzymatic function (Figure 2) that demonstrated a statistically significant increase in CS (219% of normal, *z* = 2.86, *p* < 0.01) with a statistically significant reduction in Complex IV activity (24% of normal, *z* = −2.50, *p* < 0.01). As seen in Figure 3, Seahorse respirometry of PBMCs demonstrated a statistically significant increase in Proton Leak Respiration [499% of normal, *t*(74) = 3.23, *p* < 0.001] and a statistically significant decrease in ATP-Linked respiration [82% of normal, *t*(74) = 2.39, *p* < 0.01], maximal respiratory capacity [76% of normal, *t*(74) = 3.95, *p* < 0.0001], and reserve capacity [68% of normal, *t*(74) = 4.89, *p* < 0.0001].

Whole exome sequencing identified a paternally inherited heterozygous Y1546H variant in exon 43 of unknown significance in the DEPDC5 gene (Entrez ID 9681; UniProt ID Q75140). This variant has not been published as a mutation nor has it been reported as a benign variant in the NHLBI Exome Sequencing Project database. The variant is a non-conservative amino acid substitution, which likely impacts secondary protein structure, is at a position that is highly conserved in mammals, and is predicted by *in silico* analysis to be probably damaging. This mutation was not found in his affected older sister or unaffected younger sister or mother. The variant was identified in his unaffected father, but since there has been reported to be incomplete penetrance and variable expression of DEPDC5 mutations (50, 51), this variant is considered to be potentially disease causing. Mitochondrial DNA sequencing and deletion analysis was unremarkable. Chromosomal microarray (ACL Laboratories, Rosemont, IL, USA) demonstrated a maternally inherited triplication of 12p11.1 and duplication of 15q13.3.

## Discussion

This series of two siblings with ASD outlines novel presentations of mutations in protein coding genes WDR45 and DEPDC5, which can give rise to distinct isoforms with alternative splicing and demonstrates the importance of a comprehensive evaluation of the etiology of neurodevelopmental disorders with advanced genetic testing, neuroimaging, neurophysiology, and metabolic evaluation. The two siblings had what seemed to be not dissimilar presentation of ASD at initial evaluation, but their clinical course progressed differently and demonstrated different metabolic, neurologic, and neurophysiological findings. Both had adaptive behavior composites on the VABS below 70, suggesting that they fall into the category of low-functioning ASD. Both had abnormal EEGs but the manifestation of the EEG abnormalities greatly differed with no obvious clinical manifestations in the boy and possible relation to stereotyped breathing episodes in the girl. Both manifested abnormalities in mitochondrial function, but with different patterns of mitochondrial dysfunction, which will be discussed below. Overall, these two cases provide novel presentations of ASD and highlight the importance of WES investigations.

### WDR45 Gene Mutation

We identified a novel c.795delT mutation in the WDR45 gene affecting a girl with a Rett syndrome phenotype who presented with developmental delay with seizures and late regression along with prominent teeth grinding and stereotypical cyclic breathing pattern. The WDR45 gene encodes a protein with a β-propeller architecture with multiple blades, enabling versatility. The WDR45 gene encodes a protein important for interactions necessary for appropriate autophagy (52). Prior functional analysis of mutated WDR45 has provided evidence of increasing autophagy dysfunction and resultant neurodegenerative diseases (53).

A group of disorders known as neurodegeneration with brain iron accumulation (NBIA) has been described within the past decade. Many genes have been associated with this group of disorders, including phospholipase A2, ceruloplasmin, FNS-related tyrosine kinase 1, pantothene kinase 2, fatty acid 2-hydroxylase, WDR45, and others (54). NBIA disorders have neurodegenerative changes characterized by iron deposition in the basal ganglia and substantia nigra. β-propeller protein-associated neurodegeneration (BPAN) is a recently described X-linked dominant subset of NBIA caused by *de novo* mutations in the WDR45 gene. This disorder was previously described as a static encephalopathy of childhood with neurodegeneration (SENDA) syndrome. SENDA patients initially present in early childhood with developmental delay or regression, followed by brisk cognitive decline in late adolescence or early childhood. Recent reports have suggested a broader phenotypic spectrum for mutations involving the WDR45 gene.

To our knowledge, no benign variant of WDR45 has been previously described. Although inheritance of WDR45 mutations has been described, the majority occurs *de novo* early in development. When originally described, WDR45 mutations were thought to be lethal in males, but recent reports have characterized a more severe phenotype present in males (55, 56). The initial presentation of BPAN is identified in childhood, with global developmental delay, seizures, disordered sleep, and muscle spasms with slow improvement until adulthood when cognitive decline, Parkinson disease, and global dystonia present. Corresponding imaging findings in young children are not well described. In adolescence, the characteristic iron deposition commonly become evident in the substantia nigra and cerebral peduncles on T1-weighted MRI (57).

Approximately a dozen cases of patients with WDR45 mutations have been reported to exhibit a Rett-like syndrome presentation (57–62). Most notably, in many WDR45 mutation cases, neurological regression does not occur until adolescence, although developmental delays may be prominent early on. In selected cases, autistic type regression sometimes accompanied with seizures and/or hand stereotypies have been reported, thus demonstrating the variable presentation of WDR45 mutations.

This is the first case in which the characteristic breathing pattern of Rett syndrome (periodic hyperventilation followed by apnea) has been reported, thus more tightly associating WDR45 mutations with characteristics of Rett syndrome. Interesting clobazam treatment for her abnormal EEG also improved this abnormal breathing pattern. While it is tempting to speculate that electrophysiological disturbances were driving these breathing abnormalities, it is also possible that clobazam normalized aberrant GABAergic signaling in the brainstem (63). This report supports the notion that WDR45 mutations should be considered as an underlying cause of Rett-like syndrome (57).

Having a high index of suspicion for WDR45 mutations in childhood is important as many time MRI findings are normal or only mildly abnormal, as in our case, until later childhood, adolescence, or even adulthood (59). MRI can show characteristic iron deposits despite paradoxical improvement in clinical symptoms. This indicates that levels of deposits do not necessarily correlate to phenotypic manifestation. We support the opinion of multiple other reports in expanding the phenotype of BPAN to incorporate other named syndromes (60, 64).

### DEPDC5 Gene Mutation

A potentially disease causing variant in the DEPDC5 gene was found in the brother. DEPDC5 encodes a subunit of the octameric GTPase-activating protein activity toward RAGs (GATOR) complex, which is a critical regulatory of the pathway that signals amino acid sufficiency to mechanistic target of rapamycin complex 1 (mTORC1). Inactivating mutations in GATOR1, such as DEPDC5, result in mTORC1 being hyperactive, insensitive to amino acid starvation, and hypersensitive to rapamycin (65).

A mouse model of the DEPDC5 gene mutation has found that homozygous mutations are lethal (66). Heterozygous mutations in humans have been reported to cause autosomal dominant familial focal epilepsy with variable foci, autosomal dominant nocturnal frontal lobe epilepsy, familial mesial temporal lobe epilepsy, autosomal dominant epilepsy with auditory features and infantile spasms (67), and some cases have been associated cortical dysplasia (68, 69). Like familial focal epilepsy with variable foci, DEPDC5 mutations show incomplete penetrance, which has been estimated to range from 52 to 82% (50). DEPDC5 mutations are also associated with schizophrenia, non-specific psychiatric disorders and ASD, as well as intellectual disability (50).

Besides the described case within this report, two other families with familial focal epilepsy with variable foci linked to 22q12 (the region of DEPDC5) have reported individuals with ASD features. In an Australian family, 3 of the 10 affected family members were diagnosed with ASD and intellectual disability with two of the ASD family members also having epilepsy (one parietal lobe and one temporal lobe). Two of the individuals with ASD were siblings (one with epilepsy and one without) and the other individuals with ASD was the uncle of the siblings. In a Dutch family, 2 of the 12 individuals with epilepsy were also reported to have ASD behavior, both with discharged in the frontotemporal areas (70).

This is the first case to link ASD to a mutation to DEPDC5 specifically but the fifth case to link ASD to the chromosomal region of DEPDC5. Like previous cases, this child has frontal epileptiform discharges and intellectual disability per his evaluation. With the effect of DEPDC5 on mTORC1 regulation, it would not be surprising if mutations in this gene would lead to ASD given the connection between the mTOR pathway and ASD (71). The fact that the father also had this mutation suggests that a second modifier gene or an environmental influence not known at this time must also be contributing to expression of this mutation as disease.

### Connections to Biochemical Abnormalities

It is interesting that Case 1 with NBIA shares many phenotypical characteristics and several biochemical disturbances also found in Rett syndrome, a neurodevelopmental disorder caused by mutations in the methyl-CpG-binding protein 2 gene, located on Xq28. Rett syndrome has been associated with mitochondrial dysfunction, oxidative stress, and cerebral folate deficiency. The measurement of cerebral spinal fluid 5-MTHF was low normal, suggesting cerebral problems with folate. Abnormalities in ETC function were noted with abnormally elevated activity of complex I + III in skeletal muscle and fibroblasts, complex IV in fibroblasts, and respiration in PBMCs.

Many studies have documented mitochondrial dysfunction in Rett syndrome. Biochemical measures of lactate and pyruvate have been found to be abnormally elevated in the blood and cerebrospinal fluid (72). Pathological examination has reported mitochondria with abnormal shape and morphology in skeletal muscle, fibroblasts, and both peripheral and cortical neurons of Rett syndrome patients as well as in muscle and central neurons in the Rett syndrome mouse model (73). Interestingly, despite several studies demonstrating decreases in ETC activity in patients and the mouse model of Rett syndrome (73), consistent with idea of mitochondrial disease, other reports have noted marked increases in ETC activity in the mouse model (73) and patients (14). Changes in mitochondrial-related genes associated with Rett syndrome suggest a significant upregulation of mitochondrial and nuclear encoded genes associated with ETC and general mitochondrial function (73). These changes are thought to be due to increased ETC uncoupling and decreased ETC efficiency. Similar findings were seen in our patient.

Interesting clinical and research studies from our center and others suggest that many children with ASD have novel changes in mitochondrial function similar to the ones found in Case 1. Unlike classic mitochondrial disease where mitochondrial activity is significantly depressed, individuals with ASD have been documented to have above normal ETC activity in muscle (74, 75), skin (28), buccal epithelium (38–41, 76), and brain (77). We have shown elevated mitochondrial respiration in subset of lymphoblastoid cell lines derived from children and demonstrated the repeatability of these finding (32, 78–81). This pattern of mitochondrial activity is also seen in genetic syndromes associated with ASD, including patients with Phelan-McDermid syndrome (41), 22q13 duplication (16), and Rett syndrome (14) as well as the PTEN haploinsufficient mouse model of ASD (15).

Rett syndrome is also associated with cerebral folate deficiency where the levels of 5-MTHF is below the lower limit of normal in the cerebral spinal fluid (25). However, children with ASD with cerebrospinal fluid 5-MTHF concentration in the lower normal range do respond to supplementation of folinic acid similar to our patient (82). Thus, our patient may have had similarities in abnormalities with central folate metabolism, similar to patients with Rett syndrome.

Thus, the etiology of the mitochondrial dysfunction in this case is not completely clear but given that no other specific nuclear or mitochondrial genes were found to be abnormal, it is assumed that mitochondrial dysfunction is secondary, but none-the-less, a treatment target that could improve physiological function (33). One important caveat is that this case did improve with treatment for biochemical abnormalities. However, the natural course of NBIA is for some improvement in function during childhood. Thus, the contribution of treatments cannot be certain but also cannot be ruled out. Careful clinical trials would be helpful to determine if such treatment could be helpful. Then again, this can be difficult with rare diseases where only few cases exist. Thus, other options for clinical treatment research include single-subject designs where treatments are applied and withdrawn, sometimes blindly, to determine if symptoms respond to a specific treatment.

Case 2 with the mutation in DEPDC5 demonstrated a significant reduction in complex IV on buccal swab enzymology and significant reduction in mitochondrial respiration in PBMCs, suggesting mitochondrial dysfunction. Less is known about metabolic abnormalities in patient with DEPDC5 mutations, but given that mTOR is a master metabolic regulator of the cell and can influence mitochondrial function (83), it would not be surprising if perturbation in mitochondrial function occurred because of dysregulation of mTORC1 due to DEPDC5 mutations. Indeed, further research will be needed to see if other patients with mutations in DEPDC5 or animal models of mutations in this gene also have metabolic abnormalities.

In this report, we provide evidence for a connection between WDR45 and DEPDC5 mutations and mitochondrial dysfunction. However, this evidence should be considered preliminary in the context of a single case report and such findings need to be replicated in order to determine if mitochondrial dysfunction is a characteristic of these mutations or whether other factors may have contributed our results. Indeed, we report these findings to motivate other clinicians to look for potentially treatable metabolic abnormalities in children with neurodevelopmental disorder as well as expand our understanding of the biological pathways disrupted in neurodevelopmental and genetic disorders.

## Conclusion

Neurodegeneration with brain iron accumulation provides a common diagnosis for a variety of disorders with the common causal feature of iron deposition in the basal ganglia and substantia nigra causing progressive neurodegeneration in two phases: global developmental delay in childhood with rapid onset of dementia and muscular dystonia in late adolescence or early adulthood. Case 1 and others like it certainly suggest that this new disorder should be considered in children with developmental delay accompanied by suggestive symptoms: sleep disorders, seizures, or, in this case, clinical Rett syndrome phenotype.

Likewise, this report demonstrates the expanding phenotypes that might be associated with DEPDC5 and provides some underlying biological mechanisms that may advance our understanding and might be therapeutic targets. As the DEPDC5 has variable penetrance, it will be important to better understand what triggers its expression toward disease.

Early identification of the etiology of neurodevelopmental disorders with advanced genetic techniques can help expedite the diagnosis and understand the underlying cause. Information from clinical and research evaluations can help better understand the underlying medical and biological abnormalities in order to develop novel targeted therapies.

## Ethics Statement

The study was conducted under two protocols approved by the Institutional Review Board at the University of Arkansas for Medical Sciences (Little Rock, AR, USA). One protocol specifically provided permission to investigate data collected as part of clinical evaluations while the second protocol specifically addressed obtaining samples for biochemical analysis as well as developmental and behavioral testing. Parents of participants provided written informed consent.

## Author Contributions

RF obtained the clinical data. SR, SB, and SM performed the lab work. LD performed the cognitive and behavioral evaluation. MT organized the data. BB and RF drafted the paper. BB, RF, PG, and SR interpreted the data. All authors read and approved the paper.

## Conflict of Interest Statement

The authors declare that the research was conducted in the absence of any commercial or financial relationships that could be construed as a potential conflict of interest.
